# Reliever salbutamol use as a measure of exacerbation risk in chronic obstructive pulmonary disease

**DOI:** 10.1186/s12890-015-0077-0

**Published:** 2015-08-21

**Authors:** Christine R. Jenkins, Dirkje S. Postma, Antonio R. Anzueto, Barry J. Make, Stefan Peterson, Göran Eriksson, Peter M. Calverley

**Affiliations:** Department of Thoracic Medicine, Concord Hospital, University of Sydney and The George Institute for Global Health, Hospital Rd, Concord, Sydney, NSW 2139 Australia; Department of Pulmonary Medicine and Tuberculosis, University of Groningen, University Medical Center Groningen, Groningen, PO Box 30001, 9700 RB Groningen The Netherlands; Pulmonary Section, Department of Medicine, University of Texas Health Science Center, and South Texas Veterans Health Care System, San Antonio, TX USA; Division of Pulmonary Sciences and Critical Care Medicine, National Jewish Health, University of Colorado Denver School of Medicine, 1400 Jackson Street, K729, Denver, CO 80206 USA; StatMind, Medicon Village AB, Scheelevägen 2, 22363 Lund, Sweden; Department of Respiratory Medicine and Allergology, University Hospital, Lund, 221 87 Sweden; Clinical Sciences Department, Institute of Ageing and Chronic Disease, University Hospital Aintree, Lower Lane, Liverpool, L9 7AL UK

**Keywords:** COPD, Budesonide/formoterol, Exacerbation, Reliever medication, Predictor

## Abstract

**Background:**

Debate exists regarding which endpoints most sensitively reflect day-to-day variation in chronic obstructive pulmonary disease (COPD) symptoms and are most useful in clinical practice to predict COPD exacerbations. We hypothesized that short-acting β_2_-agonist (SABA) reliever use would predict short- and long-term exacerbation risk in COPD patients.

**Methods:**

We performed a retrospective analysis of data from a study (ClinicalTrials.gov registration: NCT00419744) comparing budesonide/formoterol 320/9 μg with formoterol 9 μg (both twice daily) in patients with moderate-to-very-severe COPD; reliever salbutamol 90 μg was provided. First occurrence of reliever use >4 (low), >10 (medium), and >20 (high) inhalations/day was assessed as a predictor of short-term (3-week) exacerbation risk. Mean daily reliever use in the week preceding the 2-month visit was investigated as a predictor of the long-term (10-month) exacerbation risk, using intervals of 2–5, 6–9, and ≥10 inhalations/day.

**Results:**

Overall, 810 patients were included (61 % male; mean age 63.2 years; post-bronchodilator forced expiratory volume in 1 s 37.7 % of predicted). First occurrence of low, medium, or high reliever use was predictive of an exacerbation within the following 3 weeks; exacerbation risk increased significantly with increasing reliever use. Mean reliever use over 1 week was predictive of long-term exacerbation risk. Patients with mean use of 2–5, 6–9, and ≥10 inhalations/day exhibited 21 %, 67 %, and 135 % higher exacerbation rates, respectively, in the following 10 months, compared with <2 inhalations/day. Budesonide/formoterol was associated with lower short- and long-term exacerbation risk than formoterol in all reliever-use groups.

**Conclusions:**

SABA reliever use is a predictor of short- and long-term exacerbation risk in moderate-to-very-severe COPD patients with a history of exacerbations receiving budesonide/formoterol or formoterol.

## Background

The global burden of symptoms in patients with chronic obstructive pulmonary disease (COPD) is high [1]. Under-recognition and under-treatment of COPD can have a significant impact on day-to-day activities and quality of life for patients, resulting in avoidable disease burden, activity impairment [1], and hospitalization risk [2].

It remains unclear which endpoints most sensitively reflect the day-to-day variation in symptoms in patients with COPD. Ideally, outcomes in COPD trials should reflect the real-world behavior of patients as well as the potential of treatment to influence both current disease state and future risk [3]. The forced expiratory volume in 1 s (FEV_1_) is a reproducible and responsive measurement that reflects aspects of the pathophysiology characterizing COPD. However, FEV_1_ impairment is only weakly related to overall patient well-being [4, 5], and other endpoints are needed that are easier to obtain in routine clinical practice and that reflect the day-to-day impact of COPD on the patient, in order to guide treatment decisions [6, 7]. New symptom-based questionnaires address this need, but they may neglect the important information patients report during routine office visits or in surveys accompanied by objective measures of symptom impact [8]. An example of this type of information is the frequency with which patients use reliever medication to control symptoms experienced in the real world.

In asthma trials, the use of short-acting β_2_-agonists (SABA) as reliever medication to decrease symptoms has been accepted as a measure of day-to-day asthma control [9]. This outcome is included in widely used asthma control questionnaires as a surrogate measure of symptom frequency and a reflection of treatment efficacy [10, 11]. Until relatively recently, SABAs were prescribed to COPD patients as background maintenance therapy 1–4 times per day and, traditionally, their frequency of use has not been considered to reflect the variability of symptoms and patients’ responses. With the widespread use of long-acting inhaled bronchodilators as first-line maintenance medication in COPD [12], SABAs are now seen as reliever medication in COPD [13]. Thus, by analogy with asthma, reliever use in COPD may be a sensitive marker of symptom variability [14] and of the extent to which an intervention improves symptom control. Conversely, since increased reliever use is an indicator of sub-optimal control in asthma, it could have a similar significance in COPD, reflecting worsening symptom control or an impending exacerbation. Evidence already exists to show that, compared with as-needed SABA use, regular SABA use is not associated with additional benefit across a range of clinical and functional outcomes in COPD [15].

We hypothesized that more SABA taken as reliever medication would predict increased short- and long-term risk of exacerbations in patients with COPD, and tested our hypothesis retrospectively by analyzing reliever use collected by electronic diary recording in patients who participated in a clinical trial of budesonide/formoterol (BUD/FORM) or formoterol (FORM) [16].

## Methods

This retrospective analysis was undertaken on data collected in a study (ClinicalTrials.gov registration: NCT00419744) comparing fixed-dose combination BUD/FORM with FORM monotherapy, in which electronic diaries were used to record as-needed SABA reliever use administered using a pressurized metered-dose inhaler (pMDI) [16]. The study evaluated the effect of maintenance treatment with BUD/FORM or FORM on COPD exacerbations, defined as COPD worsening leading to oral corticosteroid therapy and/or hospitalization, in patients with moderate-to-very-severe COPD [12] who had a history of one or more exacerbations in the previous year. The study protocol was approved by an institutional review board for each of the clinical sites and written informed consent was obtained from patients or guardians before any study procedures were initiated. The study was conducted in accordance with the Declaration of Helsinki, Good Clinical Practice and applicable local regulations.

### Study design and methods

The full methodology of this trial has been published previously [16]. Following a 2-week run-in period, current COPD medications were discontinued and eligible patients who met inclusion criteria were randomized to twice-daily BUD/FORM pMDI 160/4.5 μg × 2 inhalations (320/9 μg), BUD/FORM pMDI 80/4.5 μg × 2 inhalations (160/9 μg), or formoterol dry powder inhaler (DPI) 4.5 μg × 2 inhalations (9 μg). Patients were provided with the SABA salbutamol (albuterol) 90 μg × 2 inhalations via a pMDI to administer as reliever medication throughout the study. Medications were not escalated during the trial; however, medications allowed during a COPD exacerbation were oral corticosteroids, single injection parenteral corticosteroids (not depot formulations), xanthines, and inhaled or nebulized ipratropium or β_2_-agonists. Symptoms, and morning and evening peak expiratory flow, were measured daily prior to the administration of the morning and evening dose of study medication. Use of reliever medication was recorded in the morning and evening as number of inhalations.

Patients receiving twice-daily BUD/FORM pMDI 160/4.5 μg × 2 inhalations (320/9 μg) and formoterol dry powder inhaler (DPI) 4.5 μg × 2 inhalations (9 μg) were included in the present analysis. Patients receiving the lower dose of BUD/FORM were excluded from the present analysis as it is not a registered product.

### Statistical analysis

We assessed two risk profiles for SABA use and exacerbations.

#### Short-term exacerbation risk (21 days)

The short-term exacerbation risk was evaluated as the relationship between a patient reaching a certain threshold of reliever use in a single day and the probability of having an exacerbation in the next 21 days. This short-term risk was described by analysis of time to first exacerbation after the first time a patient used more than the specified number of SABA inhalations. This was presented descriptively using Kaplan-Meier graphs for reliever use thresholds of >4 (low use), >10 (medium use), and >20 (high use) inhalations in a single day for both treatment groups, with ≥0 inhalations (i.e. all patients) as a reference group. Both the reliever use thresholds and 21-day time period were defined empirically; the reliever use thresholds broadly reflected use in clinical practice based on the authors’ clinical expertise, and the time period considered both the longest period of time following deviation from daily reliever use and the known evolution of COPD exacerbations [17]. The data were analyzed using a log-rank test; p-values were calculated for the comparisons of all groups and then individually against the reference group.

#### Long-term exacerbation risk (months 3–12)

The long-term exacerbation risk was evaluated as the relationship between mean reliever use during stable treatment in the week before the 2-month study visit and probability of an exacerbation occurring in months 3–12 of the study. This risk was described by analysis of the number of exacerbations for mean reliever use intervals (2–5, 6–9, and ≥10 inhalations/day: low, medium, and high reliever use, respectively), compared with mean reliever use <2 inhalations per day (infrequent). The long-term mean reliever use intervals were defined empirically to broadly reflect mean reliever use over a week in clinical practice based on the authors’ clinical expertise, and a 10-month time period was chosen to allow the longest time period between the 2-month visit and study end. We also determined long-term 10-month risk by analyzing the exacerbation rates by treatment in patients: i) reaching mean reliever use thresholds of ≥2, ≥6, and ≥10 inhalations per day; and ii) with mean number of inhalations (< or ≥) in a range from zero to 12 inhalations/day.

The analyses were performed using a Poisson regression analysis, adjusted for over-dispersion, with treatment as factor included for the first analysis. The analyses were not adjusted for additional covariates. Both analyses were presented with rates and ratios with 95 % confidence intervals (CI) and p-values.

The distribution of the frequency of patients’ reliever use on each of the study treatments was analyzed using Fisher’s exact test.

## Results

In total, 810 patients with moderate-to-very-severe COPD were included: 61 % male; mean age (range) 63.2 (40–87) years; mean (±SD) post-bronchodilator FEV_1_ 37.7 (12.1) % of predicted; 27.8 % of patients received inhaled corticosteroids before study run-in (BUD/FORM: 26.5 %, FORM: 29.0 %). Demographic and baseline clinical characteristics of patients included in this *post-hoc* analysis generally were similar across treatment groups (Table 1) [16]. Data were available for 807 patients in the short-term exacerbation risk analysis: 692, 351, and 91 patients reached the low (>4 inhalations/day), medium (>10 inhalations/day), and high (>20 inhalations/day) reliever use thresholds, respectively (patient n values are cumulative, i.e. all patients in the >20 subgroup are also in the >4 and >10 subgroups). In addition, data were available for 674 patients in the long-term exacerbation risk analysis: 234, 155, and 92 patients reached the mean number of inhalations/day for inclusion in the low (2–5 inhalations/day), medium (6–9 inhalations/day), and high (>10 inhalations/day) reliever use subgroups, respectively.

**Table 1:** Baseline characteristics of patients included in this *post-hoc* analysis of the Sharafkhaneh et al. study [16]

	BUD/FORM	FORM	Total
	(*n* = 407)	(*n* = 403)	(*n* = 810)
Male, *n* (%)	262 (64.4)	229 (56.8)	491 (60.6)
Age, years (range)	63.8 (40–86)	62.5 (40–87)	63.2 (40–87)
Pre-BD FEV_1_, % predicted	33.0 (10.5)	32.4 (10.1)	32.7 (10.3)
Post-BD FEV_1_, % predicted	37.9 (11.8)	37.5 (12.4)	37.7 (12.1)
Pre-BD FEV_1_/FVC ratio, %	45.9 (11.3)	46.1 (11.1)	46.0 (11.2)
Smoking history			
Current smokers, *n* (%)	138 (33.9)	154 (38.2)	292 (36.0)
Pack-years (range)	52.6 (10–200)	52.2 (10–258)	52.4 (10–258)
Most common COPD medications before run-in, *n* (%)			
β_2_-adrenergic agonists (SABA/LABA)	320 (78.6)	321 (79.7)	641 (79.1)
Adrenergics/other drugs for obstructive airway diseases	198 (48.6)	196 (48.6)	394 (48.6)
Long-acting muscarinic antagonist	123 (30.2)	109 (27.0)	232 (28.6)
Inhaled corticosteroids	108 (26.5)	117 (29.0)	225 (27.8)
No. of exacerbations in the past 12 months, *n* (%)			
1	244 (60.0)	234 (58.1)	478 (59.0)
2	95 (23.3)	99 (24.6)	194 (24.0)
3	36 (8.8)	38 (9.4)	74 (9.1)
4	22 (5.4)	19 (4.7)	41 (5.1)
≥ 5	10 (2.5)	13 (3.2)	23 (2.8)
Mean reliever use, inhalations/day^a^	5.8 (4.6)	6.0 (4.5)	5.9 (4.6)
Patients taking ICS at baseline, *n* %	108 (26.5)	117 (29.0)	225 (2.8)

Data shown as mean (±SD), unless otherwise stated. Current smokers include habitual and occasional smokers

*BD* bronchodilator, *BUD* budesonide, *FEV*_*1*_ forced expiratory volume in 1 s, *FORM* formoterol, *FVC* forced vital capacity, *ICS* inhaled corticosteroid, *LABA* long-acting β_2_-agonists, *SABA* short-acting β_2_-agonists, *SD* standard deviation

^a^Baseline: 2-week run-in period

### Short-term exacerbation risk (21 days)

**Fig. 1:**
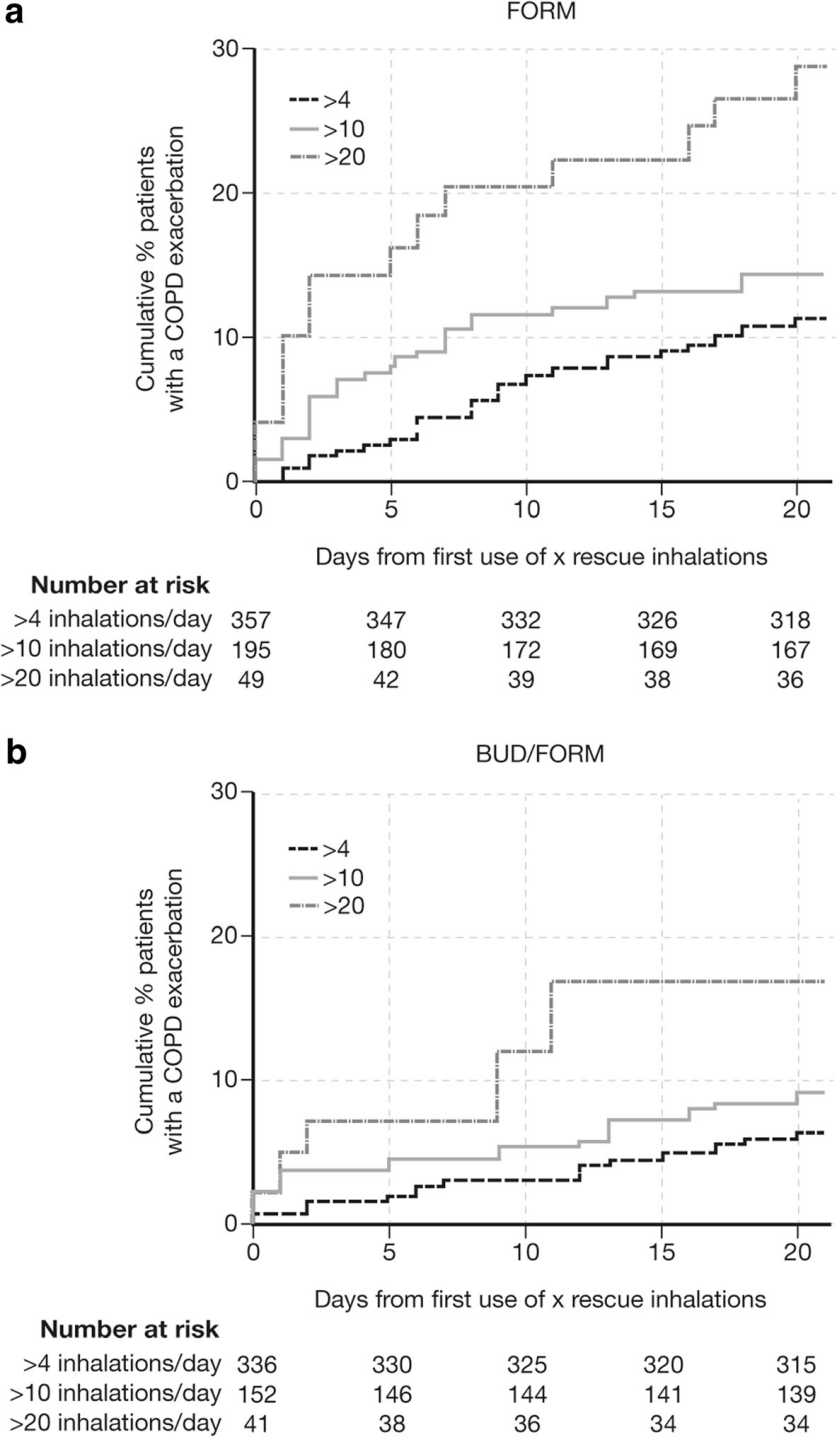
Short-term (days 0–21) exacerbation risk. Kaplan-Meier plot of patients with occurrence of an exacerbation after they used for the first time >4, >10, or >20 inhalations of salbutamol per day in **a**) FORM and **b**) BUD/FORM treatment groups. Data for 16 patients are missing from baseline to day 0. *BUD* budesonide, *COPD* chronic obstructive pulmonary disease, *FORM* formoterol

The first occurrence of reliever use beyond a certain threshold, i.e. low, medium, or high reliever use in a single day, was predictive of an exacerbation within the following 3 weeks (Fig. 1). The time to first exacerbation differed between the reliever use groups for both BUD/FORM and FORM (*p* < 0.001; Fig. 1). In addition, there was a significant increase in the risk of an exacerbation for patients who had medium and high reliever use compared with the reference group (≥0 inhalations/day) for both the BUD/FORM (*p* = 0.002 and <0.0001, respectively) and FORM (*p* = 0.02 and <0.0001, respectively) groups.

### Long-term exacerbation risk (months 3–12)

**Fig. 2:**
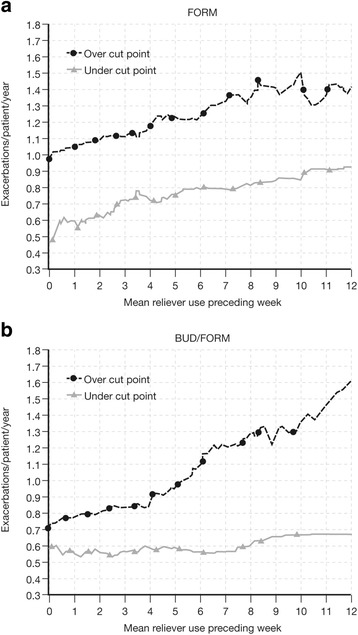
Long-term (months 3–12) exacerbation rate by reliever use thresholds. Long-term (months 3–12) exacerbation rates for patients receiving **a**) FORM and **b**) BUD/FORM with a mean number of inhalations less than, and greater than or equal to, reliever use in the range from zero to 12 inhalations/day in the week preceding the 2-month visit. *BUD* budesonide, *FORM* formoterol

**Fig. 3:**
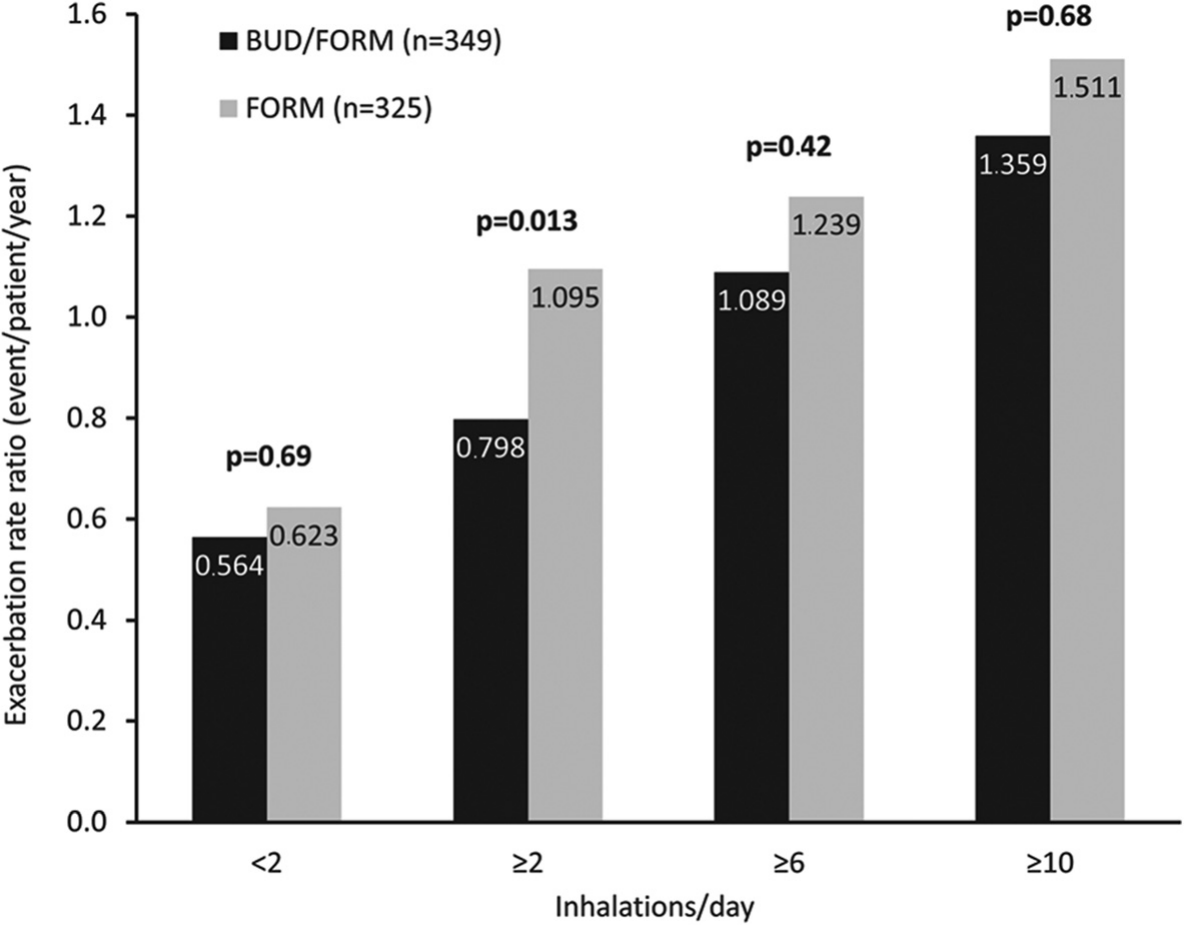
Long-term (months 3–12) exacerbation rate by treatment group. Long-term (months 3–12) exacerbation rate by treatment group, for patients reporting <2, ≥2, ≥6, or ≥10 inhalations/day in the week preceding the 2-month visit. P-values are for the analysis of BUD/FORM versus FORM. *BUD* budesonide, *FORM* formoterol

Mean daily reliever use over 1 week preceding the 2-month visit was identified as a predictor of the long-term 10-month (i.e. months 3–12) probability of an exacerbation. Patients with a mean use of 2–5, 6–9, and ≥10 inhalations exhibited 21 %, 67 %, and 135 %, respectively, greater exacerbation rates in the following 10 months relative to patients with a mean use of <2 inhalations/day; the difference being significant for the 6–9 and ≥10 groups (Table 2). For both treatments, long-term 10-month exacerbation rates were greater whenever mean reliever use exceeded thresholds, in the range from 0–12 inhalations/day in the week preceding the 2-month visit (Fig. 2). In addition, there were more infrequent reliever users in the BUD/FORM group than FORM group and, consequently, fewer patients with ≥2, ≥6, or ≥10 inhalations of reliever medication per day; this was most apparent at higher reliever use (Table 3). Patients randomized to BUD/FORM had a lower risk of exacerbations than the FORM group (Fig. 3). Patients with higher mean daily reliever use in the week preceding the 2-month visit had a higher rate of exacerbations during months 3–12 than those with lower mean daily reliever use, over a range of cut-points (Table 2 and Fig. 3). This was true for both BUD/FORM and FORM recipients.

**Table 2:** Long-term exacerbation rate. Long-term (months 3–12) exacerbation rate in all patients, based on cut-points of ≥2, ≥6, and ≥10 reliever inhalations/day in the week preceding the 2-month visit

Mean reliever use (inhalations/day)	Exacerbation rate^a^ or ratio	95 % CI	*P*-value
<2	0.596	(0.470, 0.756)	
2–5	0.724	(0.594, 0.882)	
6–9	0.996	(0.806, 1.230)	
≥10	1.403	(1.115, 1.766)	
2–5 versus <2	1.214	(0.891, 1.654)	0.22
6–9 versus <2	1.670	(1.215, 2.296)	0.0016
≥10 versus <2	2.353	(1.687, 3.282)	<0.001

*CI* confidence interval

^a^Rates are normalized for 10 months and expressed as events per year. Analysis adjusted for treatment effects

**Table 3:** Number of patients reporting <2, ≥2, ≥6, or ≥10 inhalations/day in the week preceding the 2-month visit by treatment group

Mean reliever use (inhalations/day)	BUD/FORM (*n* = 349)	FORM (*n* = 325)	*P* value^a^
Patients with cut-point inhalations/day, *n* (%)			
<2	114 (32.7)	79 (24.3)	0.017
≥2	235 (67.3)	246 (75.7)	0.017
≥6	111 (31.8)	136 (41.8)	0.007
≥10	27 (7.7)	65 (20.0)	<0.001

Patient n values are cumulative (i.e. all patients in the ≥10 group are also in the ≥2 and ≥6 groups) and represent the number of patients remaining in the analysis for the week before the 2-month visit

*BUD* budesonide, *FORM* formoterol

^a^P-values are for the analysis of BUD/FORM versus FORM

## Discussion

In this analysis, the daily number of inhalations of SABA needed for relief of symptoms was a predictor for future exacerbations in patients with COPD and a history of exacerbations in a study of combination BUD/FORM as maintenance therapy. SABA use predicted both short- and long-term risk of exacerbations. High SABA use in a single day was a predictor of the short-term probability of exacerbation in the following 21 days, whereas the average daily SABA use over 1 week of stable maintenance treatment was a strong predictor of an exacerbation in the following 10 months. This was true for patients taking BUD/FORM and FORM alone; however, patients treated with BUD/FORM had a lower short- and long-term risk of exacerbation compared with FORM monotherapy.

Although a relationship between as-needed SABA use and exacerbations in patients with COPD has long been suspected by clinicians, to our knowledge this is the first systematic investigation demonstrating this relationship. Indeed, although an increasing number of studies testing the efficacy of long-acting bronchodilators have used this index as an objective measure of the impact of treatment and a surrogate marker for breakthrough symptoms [18–21], none have specifically analyzed the data in relation to the predictive capacity of as-needed SABA use in this context. In this analysis of data from a randomized, controlled study, we showed that as-needed SABA use in COPD patients can predict exacerbation risk over the short (≤21 days) and long (≤10 months) term, if patients are taking appropriate doses of long-acting β_2_-agonist either alone or in combination with an inhaled corticosteroid. Our data suggest that for COPD, as in asthma [22], reliever use may be implemented as an important parameter for disease stability and future exacerbation risk. If our results are confirmed in other studies, it may be useful for clinicians to monitor patterns of reliever medication use so that they can identify patients at risk for an exacerbation, take steps to prevent the occurrence of a COPD exacerbation and ensure that when these events do occur, they are more rapidly identified and effectively treated. Similarly, patients may be educated to recognize increasing reliever use as a warning sign for an exacerbation and to take steps early to prevent further deterioration.

While we consider these findings to be robust for this group of patients with moderate-to-very-severe COPD and a history of exacerbations, further studies in patients with milder disease are needed before the predictive capacity of the use of SABA as a reliever can be generalized to all symptomatic patients with COPD. Ideally, for implementation in clinical trials, the findings should be confirmed in other large datasets in which similar instructions have been given to participants, with records of SABA use kept in a comparable way throughout the study. In addition, further studies would enable assessment of the degree of individual variability and clarify the ideal reliever use cut-points in predicting COPD exacerbations. We wished to validate our findings in a larger study population receiving similar BUD/FORM and FORM doses and recording reliever use, using data from five randomized controlled trials of BUD/FORM [17, 23–26], including the use of BUD/FORM in combination with a long-acting muscarinic antagonist [17]. However, methodological differences between the studies, specifically a mix of methodology (paper versus electronic diaries, different study duration and devices), meant that this analysis could not be undertaken in a suitably rigorous manner.

The reliever use categories in the present analysis were defined empirically to broadly reflect use in clinical practice based on the authors’ clinical expertise, and future analyses should validate reliever use categories to identify specific thresholds that may predict future exacerbations. However, even in the absence of further validation, measures of SABA reliever use could be incorporated into risk prediction tools for exacerbations to initiate early treatment and potentially mitigate an upcoming exacerbation. We have previously developed a risk prediction tool (SCOPEX) based on a range of demographic and baseline parameters from a pooled database of BUD/FORM COPD studies; data from this tool showed that higher mean daily reliever use was a dominant predictor of a COPD exacerbation in the next 6 months [27]. In agreement with the current analysis, the risk prediction tool showed that FORM was associated with a higher exacerbation risk than BUD/FORM.

As with the original study [16], it is unclear whether discontinuation of inhaled corticosteroids in those patients who received FORM alone contributed to worse exacerbation outcomes compared with those receiving BUD/FORM. Long-acting β_2_-agonist monotherapy is not recommended in patients with severe-to-very-severe COPD, limiting the interpretability of our data in the FORM only group. We note, however, a trend to undertake studies assessing bronchodilators alone even in severe/Group D patients, so the issue of a non-inhaled corticosteroid approach to Group D – especially those who do not exacerbate frequently – is still being discussed. In addition, we cannot verify whether the number of daily inhalations or occasions of SABA use as reliever medication was exactly as patients recorded in their electronic diaries. However, reliever use was recorded twice daily and entries were permitted only in specific time windows (morning and evening), which prevented retrospective recordings. It is well known that paper diaries may permit retrospective and fictitious entries, and even electronic diaries may suffer from recall errors [28, 29]. Optimally, electronic inhaler adherence monitoring should be used to confirm these records, but the data we analyzed were taken from a study in which this was not undertaken.

## Conclusions

SABA reliever use was a predictor of short- (≤21 days) and long-term (≤10 months) risk of exacerbations in patients with moderate-to-very-severe COPD and a history of exacerbations receiving combination BUD/FORM or FORM monotherapy. Mean reliever use over 1 week predicted exacerbation risk, and this risk increased further with a higher number of inhalations of reliever/day. These data suggest that SABA use is an important and practical outcome for assessing both current control and future risk in patients with COPD. Additional clinical trials and effectiveness studies of COPD patients with different disease severity and exacerbation history are needed to validate SABA use as a predictor of exacerbations in both clinical trials and in clinical practice.

## Abbreviations

BUD/FORM: Budesonide/formoterol
CI: Confidence interval
COPD: Chronic obstructive pulmonary disease
DPI: Dry powder inhaler
FEV_1_: Forced expiratory volume in 1 s
FORM: Formoterol
pMDI: Pressurized metered-dose inhaler
SABA: Short-acting β_2_-agonist
SD: Standard deviation
